# Acceptance and Use of Innovative Assistive Technologies among People with Cognitive Impairment and Their Caregivers: A Systematic Review

**DOI:** 10.1155/2019/9196729

**Published:** 2019-03-06

**Authors:** Björg Thordardottir, Agneta Malmgren Fänge, Connie Lethin, Danae Rodriguez Gatta, Carlos Chiatti

**Affiliations:** ^1^Department of Occupational Therapy, Prosthetics and Orthotics, Faculty of Health Sciences, OsloMet – Oslo Metropolitan University, Oslo 0130, Norway; ^2^Department of Health Sciences, Faculty of Medicine, Lund University, Lund 221 00, Sweden; ^3^Department of Clinical Sciences, Clinical Memory Research Unit, Faculty of Medicine, Lund University, Lund 221 00, Sweden

## Abstract

Cognitive impairments (CI), associated with the consequences of Alzheimer's disease and other dementias, are increasingly prevalent among older adults, leading to deterioration in self-care, mobility, and interpersonal relationships among them. Innovative Assistive Technologies (IAT) such as electronic reminders and surveillance systems are considered as increasingly important tools to facilitate independence among this population and their caregivers. The aim of this study is to synthesise knowledge on facilitators and barriers related to acceptance of and use of IAT among people with CI and their caregivers. This systematic review includes original papers with quantitative, qualitative, or mixed methods design. Relevant peer-reviewed articles published in English between 2007 and 2017 were retrieved in the following databases: CINAHL; PubMed; Inspec; and PsycINFO. The Mixed Method Appraisal Tool (MMAT) was used for quality assessment. We retrieved thirty studies, including in total 1655 participants from Europe, USA/Canada, Australia, and Asia, enrolled in their homes, care-residences, day-care centres, or Living Labs. Two-thirds of the studies tested technologies integrating home sensors and wearable devices for care and monitoring CI symptoms. Main facilitators for acceptance and adherence to IAT were familiarity with and motivation to use technologies, immediate perception of effectiveness (e.g., increase in safety perceptions), and low technical demands. Barriers identified included older age, low maturity of the IAT, little experience with technologies in general, lack of personalization, and support. More than 2/3 of the studies met 80% of the quality criteria of the MMAT. Low acceptance and use of IAT both independently and with caregivers remains a significant concern. More knowledge on facilitators and barriers to use of IAT among clients of health care and social services is crucial for the successful implementation of innovative programmes aiming to leverage innovative technologies for the independence of older people with CI.

## 1. Introduction

Age-related changes in mental and physical abilities can make independent living at home challenging. Deterioration in mobility, self-care, and interpersonal interaction and relationships has serious implications for independent living among older people [1], especially when a person has problems to remember, learn new things, concentrate, or make decisions that affect their everyday life. Cognitive impairments (CI) are increasingly prevalent in the ageing population [2] and are strongly associated with decline in activities of daily living (ADL) [3]. As the CI progresses, people become increasingly dependent on others to manage their everyday life and consequently their families and relatives (informal caregivers) are at risk of burden and stress [4]. Thus, the cost and burden of caring for older people with CI are considerable, for both informal caregivers and health care and social service (care and service) systems [5]. Efforts to reduce the societal impact of CI are needed, as well as alternative solutions to maintain independence, participation, active citizenship, and quality of the life.

Innovative Assistive Technology (IAT) is currently being developed, tested, and introduced worldwide, as an important tool to maintain independence and quality of life among community living older people with CI. This is very much in line with the European Union (EU) strategy for long-term care, which identified technologies as a key enabler for ageing in place policies and the sustainability of welfare states [5, 6]. IAT includes, e.g., sensor based surveillance and monitoring systems, mobile technology such as wearable fall detectors, and activity bracelets as well as tablets with health information or alarm functions. Indeed, the application of IAT in care and services is a rapidly changing area, in which new products and services are constantly developed and introduced at a high pace. Ambient Assisted Living (AAL) technologies, among the most promising and fast-changing types of IAT, have been categorized by Blackman and colleague [7] into different “generations,” according to how they have evolved over time. This categorization differentiates low-tech devices such as wearable alarms that only need user initiation (1^st^ generation); from systems for automatic detection of hazards (2^nd^ generation) to more complex “smart” systems integrating home sensors and wearable devices (3^rd^ generation). By now, even additional IAT not under scope of the categorization suggested by Blackman et al. [7] are emerging, e.g., social and service robots [8], in this study referred to as the “4^th^ generation.”

The use of IAT in care and services implies new lived experiences and in most cases new challenges for older people and their informal caregivers. This applies especially for older people with CI and their informal caregivers that are often old and frail themselves.

According to the literature, positive experiences of technology are prerequisites for the acceptance of any new device in general, and this may apply especially in the case of older people [9–11]. However, specific factors seem to apply to the case of older people with CI. Their ability to use technological devices in general could affect their likelihood to use IAT; however, even when older people are proficient in using a technological device such as a mobile phone [9], they may not benefit as much from other forms of more complex IAT, integrating additional components such as alarms and sensors. This could happen for example because of privacy concerns, lack of familiarity or training, and cognitive or visual impairments [10, 11].

Nonetheless, novel IAT in care could play a key role in supporting independent living of older people and could even be more important for the people with CI as it may potentially reduce their dependence on others and promote their autonomy and independence [12]. The implementation of IAT-based is, however, a multifaceted process that affects both older people themselves and their informal and formal caregivers, and the outcome is not always predictable. In this process, acceptance (the intention to use technology [13]) and adherence (the actual use after acceptance [14]) are two important dimensions to be addressed if a successful outcome is to be secured.

A recent review focusing on usability and acceptability of technology among people with mild cognitive impairment and dementia shows that a wide range of IAT is already available for this target group, e.g., digital calendars and Global Positioning System (GPS) [15], but that studies in this area remain contradictory. Updated and systematized knowledge on facilitators and barriers for the implementation of IAT in the homes of people with CI could be highly relevant for the design of future home-based support strategies involving IAT. Such efforts could optimize care effectiveness and cost-efficiency [16] as well the independence, autonomy, and active citizenship among people with CI [12]. Accordingly, the aim of this study was to synthesise the knowledge on facilitators and barriers to IAT use, including acceptance and adherence to IAT, among older people with CI and their informal and formal caregivers.

## 2. Materials and Method

### 2.1. Research Questions and Search Strategy

We performed a systematic review of the published literature, using two broad research questions:

1. What facilitators and barriers are related to acceptance and use of IAT among older people with CI and their informal and formal caregivers?
2. Are there differences regarding acceptance and adherence of IAT according to the generation of the technology?

Starting from these research questions, and together with an expert librarian, we developed a detailed search strategy. We used the PICO framework [17] as reference (excluding the C=comparison since this did not apply to our study); i.e., we limited our search to articles fulfilling the following inclusion criteria:

- P (Participants): studies enrolling people 65 years and older with any form of CI, and/or their informal and/or formal caregivers.
- I (Interventions): studies evaluating interventions using IAT exclusively or predominantly.
- O (Outcomes): studies addressing acceptance, adoption, attitude, perception, and use of the IAT based intervention, as either primary or secondary outcome.

We aimed to include articles with quantitative, qualitative, and mixed methods designs from all disciplines, with no specific restriction of study design or setting. We excluded studies addressing assistive devices, e.g., walkers, wheelchairs, and hearing and visual aids, which are considered as part of routine health care interventions and most often need a prescription or individual adaptation.

Peer-reviewed articles of primary studies fulfilling the inclusion criteria were searched in the following electronic databases: CINAHL; PubMed; Inspec; and PsycINFO, written in English and published between 2007 and 2017. Commentaries, editorials, and conference papers were excluded, together with effectiveness studies addressing only clinical benefits of using IAT, unless the abstract indicated availability of results related to our outcomes of interest. Likewise, we excluded studies focusing only on deployment, effectiveness, gaming, and safety and studies where caregivers only were involved as proxy-respondents of people with CI. Full account of the literature search strategy is given in the Appendix.

### 2.2. Article Selection

**Figure 1:**
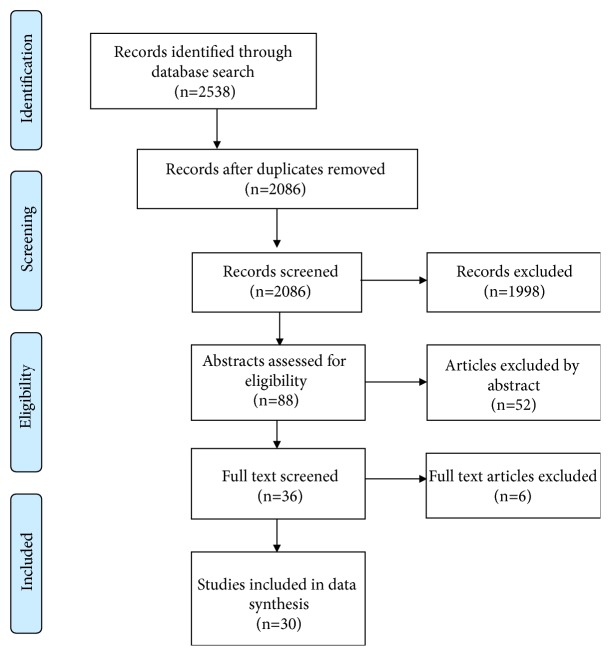
Flow diagram of the article selection process.

One of the authors (DR) independently performed the literature search and then, in parallel with the first author (BT), reviewed the titles for eligibility. The initial titles search resulted in 2538 titles out of which 452 were identified as duplicates. DR and BT separately performed a screening process based on the titles of the remaining 2086 articles. Their results were cross-checked by a third author (CC) in order to finalize a list of eligible articles to include. This resulted in 88 potentially eligible abstracts. The abstracts retained were analysed by BT according to the research questions, in order to obtain the final list of full-text papers to be reviewed. After the analysis of the abstracts, 52 of them were excluded, as they did not fit the aim of this review. Thirty-six full-text papers were then analysed, to ensure that the studies addressed actual use of the technology, as opposed to merely investigate attitudes towards possible use, resulting in a final number of 30 papers included for the full review (Figure 1).

### 2.3. Data Synthesis and Quality Assessment

Data extraction and synthesis were performed using a Summary of Findings (SoF) table, designed according to the aim of the study. The SoF table summarizes data on study context, outcomes, sample characteristics, design and type of data, characteristics of technology, and main results in terms of acceptability and adherence. In order to develop an understanding of whether acceptance and adherence were related to different generations of technology, the categorization proposed by Blackman and colleagues [7] was utilized for data synthesis. The quality of the included papers was evaluated using the Mixed Methods Appraisal Tool (MMAT), revised version [18]. This assessment tool applies different quality criteria for different study designs, thereby taking the unique characteristics of each design into consideration. In order to carry out an objective assessment of the study quality, two authors (BT and AMF) evaluated each paper separately. Disagreements between the authors (n=4 papers) were solved by means of discussions between the two until they reached consensus.

## 3. Results

### 3.1. Participants and Study Designs

The design and quality of studies according to the MMAT are presented in Table 1, which includes also more details on participants and type of data. Facilitators and barriers related to acceptance and adherence are presented in Table 2, including details on type of technology and outcomes.

A total of 1655 individuals participated in the 30 included studies (Table 1). They were people diagnosed with mild cognitive impairment (MCI), or advanced or severe dementia or Alzheimer Disease (AD), their formal and/or their informal caregivers. The included studies were performed in Europe (22), USA/Canada (5), Australia (2), and Asia (1). The studies were conducted in people's own home (20), formal residence (8), day-care centre (1), and Living Lab (1). One study was conducted in both home and formal residence. Four studies addressed the use of social robots and could thus not be categorized according to Blackman et al. (7), while the IAT in all other studies could be classified into 1^st^, 2^nd^, or 3^rd^ generation. The IAT included in the four remaining studies thus were categorized as 4^th^ generation.

Eight studies had a qualitative design, while 22 applied a quantitative or mixed methods design: one randomized controlled trial, three nonrandomized trial, eleven observational studies using quantitative measures only, and seven studies using a mixed methods design. Out of the 30 studies included, n= 20 had a dyads-based approach, i.e., involved people with CI and informal or formal caregivers, while the remaining 10 studies included either people with CI (6), formal (3), or informal caregivers (1) only (see Table 1 for details).

### 3.2. Quality of the Papers

The papers were rated according to the MMAT [18] (Table 1). Ten papers were rated as high-quality studies (^*∗∗∗∗∗*^), meeting all five quality criteria. Eleven papers were rated with four stars (^*∗∗∗∗*^), meeting 80% of the quality criteria; three articles met 60% of the criteria (^*∗∗∗*^), two met 40% (^*∗∗*^), and three studies met only 20% of the quality criteria (^*∗*^). One article received no star, i.e., met none of the quality criteria. This study was the only randomized controlled trial (RCT) included.

### 3.3. Acceptance and Adherence to IAT Use

Twelve studies presented results on IAT that were both accepted and used by the participants [19–21, 23, 27, 30–33, 42, 43, 45]. In eleven studies, the IAT was accepted but not used in the following implementation period, therefore resulting in poor adherence [24, 29, 34–38, 41, 44, 46, 47]. The main facilitators identified were ease of use, familiarity with technology, improvement of care, low technical demands, and personalized fit of IAT to daily routines. In addition, enjoyment, possibilities for new interactions, and feelings of safety also motivated the participants to use IAT. Moreover, how and when the IAT was introduced as well as the provision of support before and during the implementation were highly relevant for acceptance and adherence of the IAT (see Table 2 for details).

Regarding the barriers, the main factors hindering adherence to IAT were the participants' lack of experience of technology in general, and the age of the person using the technology [24]. This affected time-use and increased the occurrence of errors significantly [24, 29]. Other issues affecting adherence were the needs for further development of the technology [29] and that more time was necessary for the users to learn how to use IAT in order to make adherence successful [29, 34, 44, 46]. The participants had to be motivated and encouraged to make adjustments to everyday routines, and to trust their own capacity to use the technology [35]. Likewise, an immediate recognition of the benefits of IAT facilitated acceptance. In some cases users did not even mind being monitored by IAT if this was understood as a useful strategy to allow their physician to provide a better care [19].

Some studies [46, 47] also indicated the need for transparent and easy-to-understand information feedback for increasing the perceived efficiency of the technology and the need for a personalized fit between the technology and preferences of the participants [36]. Conversely, lack of clarity and feedback from the technology conveyed uncertainty, hindering acceptance and adherence. Other contextual factors potentially influencing the use of IAT include mobile network issues and internet-access (see [36] for details). User interfaces (i.e., how the technology looks like) are also of great importance. In this respect, one of the studies showed that only 1/3 of the participants were satisfied with the “look and feel” of the IAT [37]. It was found that caregivers have a significant role in the process of IAT implementation among people with CI. While some informal caregivers were less anxious after accepting to use IAT, others reported a decrease in their quality of life [38]. It was stated that IAT should support the caregivers and not replace them [21]; i.e., using only technology to monitor their health was not an option. The caregivers anticipated a reduction of the burden of care when IAT was implemented; however, stress increased when this was not the case [38]. Lastly, electricity cost was a barrier for use [41]. Summing up, our results show that when the IAT prompted safety and freedom and enhanced autonomy for people with CI [42–44], as well as relief and less worry for the caregivers [45] it was accepted and adhered to.

Seven studies reported that technology was neither accepted nor adhered to [22, 25, 26, 28, 39, 40, 48]. The study participants explained that this lack of acceptance and use was to be ascribed to their own lack of skills, fear of mistakes or being replaced, staff irritation, or fear of being replaced or due to an intrusive design, e.g., a big watch on a frail arm [40]. When IAT failed to correspond with the participants' identity and needs, their interest in using the device faded [28]. From a technical perspective, there were concerns in terms of data leakages and/or problems with the display of information on the screen, which led to the staff not trusting the IAT [39]. With IAT still under development, discrepancy between expectation and actual function may lead to nonacceptance and nonadherence [22, 25, 26]. More specifically, several barriers to robot-acceptance were identified, including older people's uneasiness with IAT, feeling of stigmatization, and ethical and societal issues associated with robot use [48].

Regarding clinical factors, the progression of the disease, and the onset of more severe symptoms, was found to negatively affect adherence to IAT [28] indicating the need for regular follow-ups to adapt the IAT to the changing needs of the client [30, 31], as well as to those of the formal and informal caregivers [32, 33]. When IAT does not correspond with the participants' expectations, their interest in using it faded [28]. For example, within an experiment testing a remote monitoring system in a nursing home, when the formal caregivers lost trust in the technology, they continued to perform physical visits to the residents [39]. In this respect, one of the reviewed studies [23] describes how the struggle with imperfect systems might end up in a success when the participants felt their attitude from fear of losing control to perceived increase in control.

In relation to the typology of IAT evaluated, we found that the majority of the IAT included in the studies could be categorized as 3^rd^ generation IAT (n=16), while five studies related to 2^nd^ generation IAT, and five studies included 1^st^ generation IAT. The largest proportion of studies demonstrating acceptance and adherence of the IAT targeted 3^rd^ generation IAT, while IAT belonging to the 2^nd^ generation were less accepted followed by the 1^st^ and 4^th^ generation of IAT (see Table 3). When it comes to use of robots in health care, our results show that the users had generally low interest to use the robot, as well as negative attitudes toward and negative images for this type of devices [24, 26, 48]. The users simply did not perceive it as useful in their daily life, although they found it easy to use, amusing, and not threatening. Direct experience with the robot did not change the way the participants rated robots in their acceptance questionnaire [48]. Personal aspects as not feeling comfortable with technology, feelings of stigmatization, and ethical and societal issues concerning robot use need further scrutiny to ensure quality in implementation of IAT [49].

## 4. Discussion

To the best of our knowledge, this review is the first attempt to systematically identify and evaluate primary studies that evaluate both acceptance and adherence to IAT among people with CI living at home, addressing also the specificity of different IAT-generations.

Our findings well represent the complexity of the two outcomes of interests: many barriers and facilitators to acceptance and adherence of IAT have been identified, each requiring duly consideration for successful implementation of IAT among people with CI and their caregivers.

From an overall perspective, difficulties and challenges in IAT research can be related to the individual technology users (micro level), the organizational processes and systems (meso level), and the national policy context (macro level) [50]. Most of the results found in our review are related to the individual user level.

One of our main findings is the importance of how the benefits of the technology are communicated first and perceived then, to the older people with CI and their formal and informal caregivers. Communication indeed seems to be an important prerequisite for acceptance and use of IAT among them. This is in line with previous research which has demonstrated that technology is not adopted at all or is soon abandoned after a short while, when end users do not perceive an immediate advantage [51]. A previous review by Peek et al. [14], targeting the general old population, provides partially overlapping results. It showed that the most important factors for acceptance of IAT were a perceived need for the technology and the expected benefits of its use. Our review integrates this knowledge, by suggesting the importance of a correct matching between expectations before implementation and the actual benefits of the technology following the initial use. Mismatch in this respect can hinder a successful implementation as consequence of the users' disappointment. Disillusion might then open the way to other factors opposing acceptance, such as perceived stigma, thus leading to the failure of the intervention [51, 52].

Our results suggest that further investigation on the mid- and long-term adherence to IAT among people with CI is needed, as many reviewed studies failed to address this perspective in the study design, i.e., had no follow-up or short time-span. Solid scientific results on postimplementation adherence are actually lacking. The lack of prospective data is particularly relevant in the context of care for people with CI, in which the “time factor” is critical. Indeed, we found that, to achieve a higher adherence, IAT needs to be introduced early in the course of the disease. Follow-up measures and adjustments for this target population are of paramount importance. As Holthe and colleagues [15] pointed out, the technology should be introduced at “the right time” and the “window” for implementation may be short in most cases. The need for adjustments has been previously underlined [53], and studies [54] have even suggested the need to create autoprompting systems that provide specific, personalized, and flexible prompts to the users. Coherently with this, our review stresses the need for personalization of technology around users' needs. The design of IAT-based interventions must consider the needs of the person with CI and the caregivers, e.g., their capabilities, preferences, and habits. In particular, our analysis underlines the importance of the caregiver role. This is in line with the results from Peek et al. [14] showing that a committed caregiver is vital throughout the technology implementation process.

With respect to the most recent technologies, such as the robots, the potential barriers for acceptance found in our review are in line with those described by Wu and colleagues [55]. The clients need to be motivated to use 4^th^ generation IAT and to understand how they can actually benefit from them before they are willing to accept and adhere to its use. This is an interesting finding for further development of service based, e.g., on social robots in older people care. Nonetheless, studies of acceptance and adherence to these new technologies in health care are still scarce [56]. Interestingly, our review reflects the fast development of this technological field, as the older study, published 2014 [48], evaluating the use of robot was conducted in a Living Lab settings, while the newest, published in 2017 [31], was performed as action research for three years in older people's own home. Moreover, the higher acceptance rate of the 2^nd^ and 3^rd^ generation technology might reflect the fact that in most cases these tech-generations were substantially similar to the first generation, being based on the same devices made more intelligent thanks to a new software. These might have facilitated acceptance among users. On the contrary, the 4^th^ generation is radically new, as it is based on new devices such as robots, with which the users are not familiar anymore. This suggests that the further development of the technologies has brought forward even more evidently the need of studies aimed at understanding acceptance among people with CI.

### 4.1. Potential Limitations of the Study

The search strategy was prepared in cooperation with a university librarian. Given the multidisciplinary of the study, the revision of abstract was particularly demanding. We found a broad diversity among the studies included as well as among the journals they were published in. The quality assessment according to the MMAT further highlighted the heterogeneity of the studies. In addition, our results are based to a minimal extent on evidence generated from randomized controlled studies. These studies are difficult to perform in this population, e.g., due to drop-out; therefore data on technology acceptance and adherence in this context was extremely difficult to retrieve.

## 5. Conclusion

Summing up, our findings show that IAT-based interventions can be accepted and used by people with CI and their caregivers. Therefore, they have the potential to compensate for functional decline, i.e., to facilitate everyday activities for several months, despite steady progression of the disease. Given their possible impact of impairment on quality of life and health, such results are promising. It is obvious that technology design and effects need to satisfy the expectations of people with CI and their caregivers. Taken together, our findings indicate a need for more individually designed IAT. Most of all, people with CI and their formal and informal caregivers need to be motivated to use IAT, i.e., understand how they can benefit personally before they are willing to accept and adhere to its use. Since most of the studies found showed that IAT was accepted by the users at the baseline assessment, our results point also to the importance of addressing adherence to IAT among people with CI in the mid- and long-term run. Such studies would be useful for the future implementation of large-scale IAT-based interventions.

## Figures and Tables

**Table 1:** Description of study details and design.

First author, title. MMAT -design/ score^1^	Study design, duration and participants (n, age)^2^	Type of data
*(1) Boise et al 2013 [19]* Willingness of older adults to share data and privacy concerns after exposure to unobtrusive home monitoring. 3/*∗*	Cross sectional survey after monitoring for 1 year in clients homes (n=119), mean age 83 years. Groups: Cognitively Intact n= 92 Cognitively impaired n=27 (MMSE≤24)	34 questions on e.g. computer use, attitudes about unobtrusive monitoring and monitoring of computer use, attitudes about sharing monitoring information with one's family or doctor, and concerns about privacy or security.

*(2) Cahill et al 2007 [20]* *“It gives me a sense of independence” *– Findings from Ireland on the use and usefulness of assistive technology for people with dementia. 5/*∗∗∗∗*	Semi-structured questionnaire after 3 months of use in clients homes, client/family caregiver dyad^3^ (n=40), 60-90 years old.	Baseline and follow-up data on use and usefulness of the product both from the individual's perspective along with from the perspective of the primary family caregiver.

*(3) Cavallo et al 2015 [21]* An Ambient Assisted Living Approach in Designing Domiciliary Services Combined With Innovative Technologies for Patients With Alzheimer's Disease: A Case Study. 4/*∗∗∗∗*	Experimental study at home and in residential living, client/formal caregiver dyad (n=30), mean age of client 84.5 years.	Interviews with 15 *socio-medical operators* before and after the experimentation phase. Information on study duration not provided

*(4) Chen et al 2012 [22]* Exploring functions of the lost seeking devices for people with dementia. 4/*∗∗∗∗*	User-centred design for 12-36 months among family – or formal caregivers (n=37), mean age 58.8 years.	In depth interview and survey on experiences and requirements of caregivers.

*(5) Engström et al 2009 [23]* Staff members perceptions of a ICT support package in dementia care during the process of implementation. 1/*∗∗∗∗∗*	Descriptive study for18 months at a residential home among formal caregivers (n=14), 25-56 years old.	Interviews in groups, once before the new ICT, twice during its implementation and once after.

*(6) Granata et al 2013 [24]* Robot services for elderly with cognitive impairment: Testing usability of graphical user interfaces. 4/*∗∗∗∗*	Usability testing comparing two groups - one trial only in clients homes (n=22), mean age 76.5 years. Groups: Elderly with MCI (n=11) Elderly cognitively healthy (n=11)	Performance measures (task completion time and number of errors) were collected

*(7) Hattink et al 2016 [25]* The electronic, personalizable Rosetta system for dementia care: exploring the user-friendliness, usefulness and impact. 2/-	Controlled trial with pre- and post-test measures in clients homes for 0-8 months, among clients, family- and formal caregivers (n=80, 32+42+6) mean age of clients 79.5 years.	Self-developed semi-structured questionnaires

*(8) Hebesberger et al 2017 [26]* A Long-Term Autonomous Robot at a Care Hospital: A Mixed Methods Study on Social Acceptance and Experiences of Staff and Older Adults. 5/*∗∗∗∗*	Mixed-method design; 15-day-trial following a 5-day-pilot testing phase at a care-hospital among formal caregivers (n=70).	Observations (12 h), questionnaires and in-depths interviews(n=10)

*(9) Imbeult et al 2013 [27]* Electronic organiser and Alzheimer's disease: Fact or fiction? 4/*∗∗∗*	Experiment at home with 2 clients aged 71 and 80 years old; 8-20 tests each <12 months.	Qualitative interviews and quantitative analysis of sensor and camera-based data on activity and behaviour.

*(10)Karlsson et al 2015 [28]* The Challenge of Coming to Terms with the Use of a New Digital Assistive Device: A Case Study of Two Persons with Mild Dementia. 1/*∗∗∗∗∗*	Explorative user study in the home of 2 clients 60 and 80 years old for 6-18 months.	Participant observations and interviews

*(11) Kerkhof et al 2015 [29]* Experiences of using a memory aid to structure and support daily activities in a small-scale group accommodation for people with dementia. 1/*∗∗∗∗∗*	Explorative study for 3 months at a residency among clients 60-80 years old and their family- and formal caregivers (n=17, 6+5+6).	Individual interviews with residents, focus groups interviews with family- and formal caregivers.

*(12) Kerssens et al 2015 [30]* Personalized Technology to Support Older Adults With and Without Cognitive Impairment Living at Home. 4/*∗∗∗∗*	Exploratory intervention for 1-2 months in 7 homes with client and family caregiver dyads (n=14) 60-88 years old.	Life Story and Care Needs interviews Engagement assessment and robot acceptability survey.

*(13) Khosla et al 2017 [31]* Human Robot Engagement and Acceptability in Residential Aged Care. 3/*∗∗∗∗∗*	Technology development and action research trial for 3 years among clients at a residency (n= 115), 65-90 years old.	Observations and questionnaires

*(14) Lazar et al 2015 [32]* Involving Family Members in the Implementation and Evaluation of Technologies for Dementia: A Dyad Case Study. 1/*∗∗∗∗∗*	Explorative use of touch screen at a residency with a client/family caregiver dyad, 86/ 60 years old.	Interview with a family member at baseline, 3 months and 6 months.

*(15) Lazar et al 2016 [33]* Evaluation of a multifunctional technology system in a memory care unit: Opportunities for innovation in dementia care. 5/*∗∗∗∗∗*	Explorative use of touch screen, at a residency with client, family- and formal caregiver (n= 16, 5+4+7) age 32-88 years,	Interviews at baseline and 6 months, with an optional interview at 3 months.

*(16) Lim et al 2013 [34]* Usability of Tablet Computers by People with Early-Stage Dementia. 4/*∗∗∗∗*	7-day trials of use of a tablet computer at home with client/family caregiver dyads (n=48), ages 34-91.	Questionnaires

*(17) Lindquist et al 2013 [35]* Significant junctures on the way towards becoming a user of assistive technology in Alzheimer's disease. 1/*∗∗∗∗∗*	Explorative usability testing for 6 months in the home with client/family caregiver dyads (n=20). Mean age of client 67 years.	Semi-structured interviews

*(18) Lindquist et al 2015 [36]* Experienced usability of assistive technology for cognitive support with respect to user goals. 1/*∗∗∗∗∗*	Explorative usability testing – 2x 6-month interventions in the home with client/family caregiver dyads (n=28). Mean age of client 69.6 years	Semi-structured interviews on expectations, interviews on experience and field notes

*(19) Magnusson et al 2014 [37]* Extended safety and support systems for people with dementia living at home. 4/*∗∗∗∗*	Intervention study with a pre-post design, 8 months at home with clients, family-and formal caregivers (n=155, 63+62+30) mean age of clients 75.7 years	Questionnaires and Extended Safety and Support (ESS) logs.

*(20) Mitseva et al 2012 [38]* Gerontechnology: Providing a Helping Hand When Caring for Cognitively Impaired Older Adults—Intermediate Results from a Controlled Study on the Satisfaction and Acceptance of Informal Caregivers. 3/*∗∗*	Controlled study in the home with client/family caregiver dyads (n=142). Mean age of client 77.4 years. Follow-up at 15 months with intermediate evaluation.	Questionnaires, interviews and structured observations

*(21) Niemeijer et al 2014 [39]* The Use of Surveillance Technology in Residential Facilities for People with Dementia or Intellectual Disabilities: A Study Among Nurses and Support Staff-Exploring the benefits and drawbacks. 1/*∗∗∗∗∗*	Ethnographic field study among nurses and support staff (n=38) using surveillance technology for 4 months at a residential home.	Field observations, formal interviews and informal conversations

*(22) Nijhof et al 2012 [40]* How assistive technology can support dementia care: A study about the effects of the IST Vivago watch on patients' sleeping behavior and the care delivery process in a nursing home. 5/*∗∗∗*	Explorative mixed-method design for 6 months at a residential home, with client/ formal caregiver dyads (n=14), 45-95 years old.	Monitor log of sleep/wake rhythm, a diary about usage, care- interventions related to the monitoring data; observations, and in-depth interviews with caregivers about implementation and usage.

(23) Nijhof et al 2013 [41] A personal assistant for dementia to stay at home safe at reduced cost. 5/*∗∗*	Explorative mixed-method design for 9 months at home with client/family caregiver dyads (n=14), 35-86 years old.	Log files, interviews with family caregivers, a focus group made up of professional caregivers, observations of project group meetings and a cost analysis

*(24) Oderud et al 2015 [42]* Persons with Dementia and Their Caregivers Using GPS. 5/*∗*	Cohort study, 36 months between in/outside the home with clients/family caregiver dyads (n= 416), 59-90 years old.	Questionnaires, semi-structured interviews, focus groups, discussion groups and home visits.

*(25) Olsson et al 2013[43]* A passive positioning alarm used by persons with dementia and their spouses – a qualitative intervention study. 1/*∗∗∗∗∗*	Qualitative intervention study 6 months, client/family caregiver dyads (n=10), 55-73 years old.	Interview text transcripts and field notes analysed using qualitative content analysis.

*(26) Perilli et al 2013 [44]* A computer-aided telephone system to enable five persons with Alzheimer's disease to make phone calls independently. 4/*∗∗∗∗*	Explorative intervention with a non-concurrent multiple baseline design, individual sessions across two groups at a daycentre, n=40, mean age 80 years old.	A social validation assessment: rate the patients' performance with the technology and with the help of a caregiver. Group 1=28 sessions; Group 2=58 sessions.

*(27) Pot et al 2012 [45]* A pilot study on the use of tracking technology: Feasibility, acceptability, and benefits for people in early stages of dementia and their informal caregivers. 4/*∗∗∗∗*	Quasi-experimental pre-post pilot study -three-month use of GPS at home/outdoor with client /family caregiver dyads (n=56), 63-73 years old.	Impression of the device on a scale ranging from 1 to 10. Several questions on the use of the device with structured response categories ranging from ‘Totally agree' to Totally disagree' and agree to disagree, respectively.

*(28) Thorpe et al 2016 [46]* Pervasive assistive technology for people with dementia: a UCD case. 4/*∗∗∗*	Controlled usability testing for one week at home with client/family caregiver dyads (n=10), 61-73 years old.	Video recordings, interaction logs, system usability scales, logbooks and interviews.

*(29) Topo et al 2007 [47]* *“I don't know about the past or the future, but today it's Friday” *– Evaluation of a time aid for people with dementia. 4/*∗∗∗∗*	Assessment study for 3 months at home with client/family caregiver dyads (n= 74), 29-99 years old	Findings from the first three months, interviews and home visits

*(30) Wu et al 2014 [48]* Acceptance of an assistive robot in older adults: a mixed-method study of human–robot interaction over a 1-month period in the Living Lab setting. 5/*∗*	Explorative mixed methods study on robot-acceptance in a Living Lab once a week for 4 weeks, n=11, 76-85 years old.	Questionnaire, semi-structured interviews, usability-performance measures, and a focus group

^1^The number/asterisk refer to design/quality according to the Mixed Method Appraisal Tool (MMAT) [18].

^2^Diagnosis is presented in comparative studies.

^3^Dyads are equally represented by a client and a caregiver, unless otherwise specified.

**Table 2:** Acceptance and adherence to innovative assistive technology (IAT).

First author, year	Type of technology	Outcome(s)	Facilitators	Barriers
Tech generation^1^				
*(1) Boise et al 2013[19]* *2*^*nd*^* generation*	Sensor technology to detect cognitive changes and other health problems.	Willingness to share health- or activity data.	Acceptance of in-home monitoring and willingness to share data with one's doctor or family members.	Concerns related to privacy or security after one year of participation.

*(2) Cahill et al 2007[20]* *1*^*st*^* generation*	The Automatic Night & Day Calendar; The Lost Item Locator; The Automatic Night Lamp; The Gas Cooker Device; The Picture Button Telephone	Use and usefulness of assistive technologies Possible refinement and financially viable on the open market.	Familiarity may influence use and usefulness. Low level of technical demands means high level of acceptance and adherence. Informal caregiver was willing to pay for useful technology	Products should be more fully refined and pre-tested on a sample of cognitively intact people before being trialled in the homes of people with dementia. High level of technical demands means low level of use/ acceptance and adherence.

*(3) Cavallo et al 2015[21]* *2*^*nd*^* generation*	A modular technological system to help caregivers monitor the health status, safety, and daily activities of patients with Alzheimer Disease.	Acceptability and usability features.	To support caregivers, not replace them, to guaranty suitability and thereby acceptance and adherence.	No information was given on the time frame of the experimental phase

*(4) Chen et al 2012[22]* *1*^*st*^* generation*	Lost seeking devices	Actual needs of the elders in using the lost seeking devices and the problems they encountered.		The choice of lost seeking device depends on the education level of the caregivers. Support in that respect is needed to overcome barriers.

*(5) Engström et al 2009[23]* *2*^*nd*^* generation*	Alarms, fall detectors, sensor-activated night-time illumination of the lavatory, and communication technology: Internet communication and additional computers.	Staff members' perceptions of an information and communication technology (ICT) support package during the process of implementation.	“Moving from fear of losing control to perceived increase in control and security” Improvements in both formal and informal care.	“Struggling with insufficient/deficient systems”

*(6) Granata et al 2013[24]* *Robot* *(4th generation)*	Social assistive robot providing grocery shopping list and an agenda application.	Usability of robot interface.	Younger participants and those with previous computer experience were faster at completing the tasks.	More errors among participants with neurocognitive disorder (NCD). Being slower at completing tasks than peers contributed to less adherence

*(7) Hattink et al 2016[25]* *3*^*rd*^* generation*	The Elderly Day Navigator; The Early Detection System; and The Unattended Autonomous Surveillance - Advanced Awareness and Prevention System.	Usefulness and user-friendliness of the Rosetta system.		The user-friendliness of the system was not rated highly. Further development is needed.

*(8) Hebesberger et al 2017[26]* *Robot* *(4th generation)*	Long-term autonomous robot able to navigate and function independently over a longer period of time without any intervention by technicians.	Usability, social acceptance.		Interacting modalities have to meet the very needs of specific end-user. Perceived utility of a robot is very much tied to its tasks and proper functioning. Social acceptance was ambivalent.

*(9) Imbeult et al 2013[27]* *3*^*rd*^* generation*	Virtual assistance system with cameras and motion sensors.	Workload reduction for prof caregivers, user satisfaction, acceptance and engagement for older people.	Positive results in terms of the satisfaction of the elderly and interaction in event handling, despite progression of the disease.	

*(10) Karlsson et al 2015[28]* *3*^*rd*^* generation*	The device consists of two parts: support memory, social contact, daily activities; and enhance the feeling of safety. Adjustable to meet the needs of the individuals using them.	Acceptance and usage of a new digital assistive device		Participant needs encompassed occupation, safety, social interaction, and memory support together with the receipt of general support. Requirement for both participants was a need to maintain their self-image. When the digital assistive device did not correspond with the participants' expectations or view of themselves, their interest in using it faded.

*(11) Kerkhof et al 2015[29]* *3*^*rd*^* generation*	Digital planning boards	To improve the use of these devices from the users' perspectives.	The majority of the residents were happy with the use and function of the memory aid.	The occurrence of errors limits ease of use and lack of knowledge on function and use among user's prevented adherence

*(12) Kerssens et al 2015[30]* *3*^*rd*^* generation*	Touchscreen computer using audio-visual programs (“shows”). Menu created based on Life Story and Care Needs interviews.	Usability, feasibility, and adoption	The technology was easy to use and significantly facilitated meaningful and positive engagement, and simplified daily lives.	

*(13) Khosla et al 2017[31]* *Robot*	Reminder on daily schedule, weather, news, date, and time. The robot can also make skype calls.	Acceptability while interacting with a social robot.	By using engagement assessment methods and robot acceptance model, the post-trial survey verified acceptance of and adherence to the interaction with social robots.	

*(14) Lazar et al 2015[32]* *3*^*rd*^* generation*	Touchscreen- a variety of applications	Perception of intervention - qualitative design.	By being aware of interests and limitations, facilitate participation and acknowledge emotions and individual barriers to adoption, and fitting technology into an establish routine, the informal caregiver was able to benefit from using the technology.	

*(15) Lazar et al 2016[33]* *3*^*rd*^* generation*	Same as above.	Perception of intervention - quantitative design.	The technology facilitated enjoyment, interactions, connections and mental stimulation.	

*(16) Lim et al 2013[34]* *3*^*rd*^* generation*	Tablet IAT	Usability of tablet as a source of leisure.	When clients were able to use the tablet computer independently, it proved to be helpful to their informal caregivers.	Adherence needs further exploration (only 7-day-in-home trial).

*(17) Lindquist et al 2013[35]* *3*^*rd*^* generation*	Mobile phone, item locator, information panel, reminder, electronic calendar, alarm, digital note taker.	What the use of IAT came to mean to these users and their significant others.	How the initial decision was made, how routines to incorporate the IAT were adjusted, whether the participants trusted the IAT, and whether the participants felt an increased sense of capacity when using the IAT.	The user has to be able to identify difficulties and needs and be motivated to become a user.

*(18) Lindquist et al 2015[36]* *3*^*rd*^* generation*	Mobile phone, item locator, information panel, reminder, electronic calendar, alarm, digital note taker	Experienced usability of features in AT to support users in desired goals in everyday activities.	Constant visible information. User's sense of control was promotional for achieving user goals.	Lack of clarity and feedback of the IAT prompted uncertainty and ineffectiveness. The users has to see the need to become a user

*(19) Magnusson et al 2014[37]* *3*^*rd*^* generation*	Extended safety and support (ESS).	Complexity surrounding the implementation of advanced electronic tracking communication and emergency response	The clients were more independent. Half of the formal caregivers considered that nearly half of their clients could remain living at home with the ESS. Informal caregivers were less stressed or anxious	Informal caregivers did not have more time for their own activities.

*(20) Mitseva et al 2012[38]* *2*^*nd*^* generation*	Platform of personalized home telecare for intelligent home support services.	The informal caregiver user acceptance satisfaction	The most successful adoption of the services can happen when they are offered as early as possible in the history of the disease	Decrease in quality of life among informal caregivers.
*(21) Niemeijer et al 2014[39]* *2*^*nd*^* generation*	Surveillance technologies.	Benefits and drawbacks of technology to support caretakers		The formal caregivers were worried about clients' safety. They need to understand and feel comfortable in using IAT to facilitate the clients' autonomy.

*(22) Nijhof et al 2012[40]* *1*^*st*^* generation*	A special watch which measured sleep/wake rhythm.	The research questions focus on the introduction of the watch, its usage and usability, the interventions that have been taken based on using the watch and the effects of the watch on the sleeping behaviour of the clients.		The IAT was described as big, clumsy and uncomfortable.

*(23) Nijhof et al 2013[41]* *3*^*rd*^* generation*	Support touch-screen.	The advantages and disadvantages of the system from the perspective of the client, informal/formal caregiver and the potentials to upscale its use.	Clients and informal caregiver reported good support of daily life activities, the system could help the client to live at home for a longer period of time, despite e.g. limited user friendliness of the lay-out.	Insufficient quality, caregiver know-how and limited involvement of informal caregivers limited usability. Electricity was considered a cost barrier.

*(24) Oderud et al 2015[42]* *3*^*rd*^* generation*	GPS Technologies.	Autonomy and independence among clients.	Increased safety for all participants. Clients maintain autonomy and continue their outdoor activities.	Half of the participants had stopped using the IAT after 3 years due to their worsening physical or mental level of functioning

*(25) Olsson et al 2013[43]* *3*^*rd*^* generation*	GPS Technologies.	Describe and explore the use and experiences of using a positioning alarm,	Previous use of technology and flexibility of the system facilitates trust in the alarm and in one own ability to use it.	

*(26) Perilli et al 2013[44]* *1*^*st*^* generation*	A net-book computer with specific software, a global system for mobile communication modem (GSM), a micro-switch, and lists of partners to call with related photos	To make phone calls independently.	All the patients learned to use the system and made phone calls independently to a variety of partners, such as family members, friends, and caregivers.	No information.

*(27) Pot et al 2012[45]* *3*^*rd*^* generation*	GPS Technologies.	Feasibility, acceptability, and effectiveness.	The majority of the informal caregivers were able to integrate the use in their daily life. The clients experienced more freedom and were less worried going out alone.	

*(28) Thorpe et al 2016[46]* *3*^*rd*^* generation*	Smartphone, smartwatch and *various applications* to offer six support features.	User-centred approach to developing and testing IAT based on off-the-shelf pervasive technologies.	Clients' motivation, personalized fit and familiarity of the technology.	Clients' motivation, personalized fit and familiarity of the technology.

*(29) Topo et al 2007[47]* *1*^*st*^* generation*	Night and Day calendars (NDC)	How the time-aid was used; and did they find it useful.	Clients' motivation and a personalized fit of the technology.	Clients' motivation and a personalized fit of the technology.

*(30) Wu et al 2014[48]* *Robot* *(4th generation)*	An indoor mobile platform with two propulsive wheels used as a generic platform and designed to ease the development of advanced robotics solutions. It can recognize and synthesize voices, and navigate in unknown environments. It also remembers appointments, manages shopping lists, plays music, and can be used as a video conference system.	To observe robot-acceptance in older adults.		Participants with neurocognitive disorder (NCD) needed more time to adjust to robot use than their cognitively intact peers. Both groups showed low intention to use the robot, as well as negative attitudes toward this device since they did not perceive it as useful

^1^ According to Blackman et al. [7].

**Table 3:** Timeline of included studies, generations according to Blackman et al. [7].

Year, title	First author	Ref.	Context	Acceptance	Adherence
*1st generation*					
2007	Cahill	[20]	Home	Yes	Yes
2007	Topo	[47]	Home	Yes	No
2012	Chen	[22]	Home	No	No
2012	Nijhof	[40]	Residency	No	No
2013	Perilli	[44]	Daycenter	Yes	Not adressed
*2nd generation*					
2013	Boise	[19]	Home	Yes	Yes
2015	Cavallo	[21]	Home+ residency	Yes	Yes
2009	Engström	[23]	Residency	Yes	Yes
2012	Mitseva	[38]	Home	Yes	No
2014	Nijemeijer	[39]	Residency	No	No
*3rd generation*					
2016	Hattink	[25]	Home	No	No
2013	Imbeault	[27]	Home	Yes	Yes
2015	Karlsson	[28]	Home	No	No
2015	Kerkhof	[29]	Residency	Yes	No
2015	Kerssens	[30]	Home	Yes	Yes
2015	Lazar	[32]	Residency	Yes	Yes
2016	Lazar	[33]	Residency	Yes	Yes
2013	Lim	[34]	Home	Yes	Not addressed
2013	Lindquist	[35]	Home	Yes	No
2015	Lindguist	[36]	Home	Yes	No
2014	Magnusson	[37]	Home	Yes	No
2013	Nijhof	[41]	Home	Yes	No
2015	Oderud	[42]	Home	Yes	Yes
2013	Olsson	[43]	Home	Yes	Yes
2012	Pot	[45]	Home	Yes	Yes
2016	Thorpe	[46]	Home	Yes	No
*Robots (4th generation)*					
2013	Granata	[24]	Home	Yes	No
2017	Hebesberger	[26]	Hospital	No	No
2017	Khosla	[31]	Residency	Yes	Yes
2014	Wu	[48]	Living Lab	No	No

**Table 4:** History of search strategies for systematic review.

Date	Search engine	Search terms	N^o^ of Results
*06/07/17*		Search terms: (i) dementia OR alzheimers OR “cognitive impairment” OR “cognitive disorders” OR “cognitive disorder” OR “cognitive decline” (ii) technology OR gerontechnology OR “smart home” OR “mobile health” OR mhealth OR telemonitoring OR monitoring OR assistive OR e-health (iii) use OR usage OR attitude OR attitudes OR perception*∗* OR acceptance OR adopt*∗* Filters: (i) Age: +65	*CINAHL * Results: 505 (examples: Alzheimer's disease, people with dementia) Results with age filter: 243 *MEDLINE (changing database in the previous interface)* Results: 1321 (examples: people with dementia, young onset dementia,) Results with age filter: 496 *PsycINFO (changing database in the previous interface)* Results: 1020 (examples: people living with dementia, younger people with dementia, Results with age filter: 427 *INSPEC (changing database in the previous interface)* Results: 240 (examples: people with dementia, early stage Alzheimer's disease) Results with age filter: Not applicable, the filter is not available

## Appendix

See Table 4.
